# Proglucagon Promoter *Cre*-Mediated AMPK Deletion in Mice Increases Circulating GLP-1 Levels and Oral Glucose Tolerance

**DOI:** 10.1371/journal.pone.0149549

**Published:** 2016-03-24

**Authors:** Sophie R. Sayers, Frank Reimann, Fiona M. Gribble, Helen Parker, Sagen Zac-Varghese, Stephen R. Bloom, Marc Foretz, Benoit Viollet, Guy A. Rutter

**Affiliations:** 1 Department of Cell Biology and Functional Genomics, Imperial College London, London, W12 ONN, United Kingdom; 2 Wellcome Trust - MRC Institute of Metabolic Science, University of Cambridge, Hills Road, Cambridge, CB2 0QQ, United Kingdom; 3 Department of Investigative Medicine, Imperial College London, London, W12 ONN, United Kingdom; 4 INSERM, U1016, Institut Cochin, 75014 Paris, France; 5 CNRS, UMR8104, 75014 Paris, France; 6 Université Paris Descartes, Sorbonne Paris Cité, 75014 Paris, France; University of British Columbia, CANADA

## Abstract

**Background:**

Enteroendocrine L-cells synthesise and release the gut hormone glucagon-like peptide-1 (GLP-1) in response to food transit. Deletion of the tumour suppressor kinase LKB1 from proglucagon-expressing cells leads to the generation of intestinal polyps but no change in circulating GLP-1 levels. Here, we explore the role of the downstream kinase AMP-activated protein kinase (AMPK) in these cells.

**Method:**

Loss of AMPK from proglucagon-expressing cells was achieved using a preproglucagon promoter-driven *Cre* (iGluCre) to catalyse recombination of floxed alleles of AMPKα1 and α2. Oral and intraperitoneal glucose tolerance were measured using standard protocols. L-cell mass was measured by immunocytochemistry. Hormone and peptide levels were measured by electrochemical-based luminescence detection or radioimmunoassay.

**Results:**

Recombination with iGluCre led to efficient deletion of AMPK from intestinal L- and pancreatic alpha-cells. In contrast to mice rendered null for LKB1 using the same strategy, mice deleted for AMPK displayed an increase (WT: 0.05 ± 0.01, KO: 0.09±0.02%, p<0.01) in L-cell mass and elevated plasma fasting (WT: 5.62 ± 0.800 pg/ml, KO: 14.5 ± 1.870, p<0.01) and fed (WT: 15.7 ± 1.48pg/ml, KO: 22.0 ± 6.62, p<0.01) GLP-1 levels. Oral, but not intraperitoneal, glucose tolerance was significantly improved by AMPK deletion, whilst insulin and glucagon levels were unchanged despite an increase in alpha to beta cell ratio (WT: 0.23 ± 0.02, KO: 0.33 ± 0.03, p<0.01).

**Conclusion:**

AMPK restricts L-cell growth and GLP-1 secretion to suppress glucose tolerance. Targeted inhibition of AMPK in L-cells may thus provide a new therapeutic strategy in some forms of type 2 diabetes.

## Introduction

Release of hormones from enteroendocrine cells in response to food transit through the gut, and the consequent activation of insulin release beyond that prompted by the rise in blood glucose alone, is responsible for the incretin effect during feeding [1,2]. L-cells make up less than 1% of the epithelial cells lining the intestinal wall, but are vital for normal physiology and energy metabolism [3,4]. L-cells are thus responsible for the synthesis and secretion of glucagon-like peptide-1 (GLP-1), GLP-2, peptide YY (PYY) and oxyntomodulin via the action of prohormone convertases (PC) 1/3 on proglucagon [5]. Although the mechanisms which trigger secretion from L-cells in response to nutrients are debated [6], roles for sodium-glucose co-transporters (SGLTs), ATP-sensitive K^+^ (K_ATP_) channels and an array of G-protein-coupled receptors have all been implicated.

GLP-1 receptors (GLP1R) are present on the pancreatic beta-cell and agonism at these receptors by L-cell-derived peptides, or by stabilised analogues such as liraglutide [7], is of considerable therapeutic interest in the treatment of type 2 diabetes (T2D). Binding of GLP-1 to GLP1R on pancreatic beta-cells triggers cAMP synthesis and downstream signalling by Protein kinase A (PKA) and Exchange Protein Activated by cAMP-2 (EPAC2), to activate insulin secretion [8,9]. Although a matter of debate [10], enhanced ATP synthesis [11], closure of K_ATP_ channels and Ca^2+^ influx may also play a role [12]. Whether the effects of GLP-1 are chiefly achieved through an action of the circulating hormone [13], or reflect an paracrine reflex loop triggered by GLP1 released in the gut [14,15], is also contested.

Released from pancreatic alpha-cells, glucagon is generated by the action of prohormone convertases (PC) 2 on proglucagon, and serves as the main anti-hypoglycaemic hormone in mammals [16]. Whilst elevated secretion of the hormone contributes to hyperglycemia in earlier stages of Type 2 diabetes T2D [17], impaired release is observed in patients living with Type 1 diabetes (T1D) and in long-standing T2D [18].

AMP-activated protein kinase (AMPK) is an evolutionarily-conserved fuel-sensitive serine/threonine protein kinase and cellular nutrient sensor implicated in the regulation of energy homeostasis [19] [20]. AMPK exists as a heterotrimeric complex comprising a catalytic α (α1and α2; encoded by *Prkaa1* and *Prkaa2*) subunit, a scaffold β (*Prkab*; β1 and β2) subunit and a regulatory *g* (*Prkag*; γ1, γ2 or γ3) subunit [21,22]. The AMPK complex is activated in response to elevated intracellular AMP:ATP [23] and ADP:ATP ratios [24]. Responses to these changes in nucleotide ratios are chiefly, though not exclusively, controlled by alterations in the susceptibility to phosphorylation by upstream kinases, notably liver kinase B1 (LKB1; STK11) and Ca^2+^ / calmodulin-dependent kinase kinase II (CaMKKII) [25], of threonine-172 in the key catalytic loop of the α-subunit [26]. We have previously shown that changes in AMPK activity are important for the regulation of both insulin [27,28] and glucagon [29,30] secretion by glucose and other nutrients. Whether this enzyme is also important in the control of hormone secretion from L-cells is unknown.

LKB1 is a tumour suppressor deleted in Peutz-Jeghers syndrome (PJS), a condition characterised by the appearance of hamartomatous polyps in the gut and increased risk cancer [31]. In addition to phosphorylating AMPK, LKB1 is also responsible for the activation for a further 12 downstream kinases of the AMPK-related kinase (AMPK-RK) family [32]. We have previously observed [33] that deletion of LKB1 from proglucagon-expressing cells, achieved by crossing animals bearing a glucagon promoter-driven Cre recombinase (iGLuCre) to mice with floxed LKB1alleles, leads to the development of large gastro-duodenal polyps, reminiscent of those seen in PJS [31]. This leads to premature death from ~ 14 weeks as a result of impaired gastric emptying. Lineage tracing revealed that LKB1 ablation caused an epithelial-to-mesenchymal transition (EMT) in LKB1 null proglucagon-expressing cells, and consequently the formation of a population of smooth muscle-like cells which expanded to form polyps.

The present study was therefore designed with two goals in mind. Firstly, to determine whether the suppressive effect of LKB1 on EMT in L-cells or their progenitors is mediated by AMPK or via other AMPK-RKs [32], and secondly to explore the role of AMPK in controlling circulating GLP-1 levels.

## Materials and Methods

### Materials

Unless indicated otherwise, reagents were purchased from Sigma (Poole, Dorset, U.K.).

### Methods

#### Generation of mice selectively lacking AMPK α1 and α2 in proglucagon-producing cells

Mice homozygous for AMPKα1^fl/fl^ were first crossed to mice homozygous for AMPKα2^fl/fl^. The resulting double heterozygous AMPKα1^fl/+^:α2^fl/+^ mice were crossed with iGluCre expressing animals [34], where ~100 kB of the 5’ flanking region of the mouse proglucagon promoter drives *Cre* expression. The latter provides efficient recombination both in L-cells and in pancreatic alpha-cells, with a minor degree of recombination also in pancreatic beta-cells [35]. The above strategy generated triple heterozygous iGluCre:AMPKα1^fl/+^:α2^fl/+^-positive mice. The latter were bred with AMPKα1^fl/^fl:α2fl^/fl^ mice to produce iGluAMPKdKO animals and further crossed to AMPKα1^fl/^fl:α2fl^/fl^ animals to generate littermate controls. As previously reported using STOP-*flox*-tdRFP reporter mice [34,35], recombination catalysed with this *Cre* deleter strain occurs in > 75% of pancreatic α cells, ~ 70% of intestinal L-cells. Low levels of recombination were also found in the olfactory bulb and hind brain [35]. All mice were kept on a C57/BL6 background.

#### Mouse maintenance and diet

Mice were housed in cages with 2–6 mice per cage in a pathogen free facility with a 12 hour light and dark cycle. Animals had *ad libitum* access to standard mouse chow diet (Research Diet, New Brunswick, NJ). All *in vivo* procedures were conducted in accordance with U.K. Home Office regulations (Animal Scientific Procedures Act of 1986, Home Office Project License number PPL 70/06608, holder Dr Isabelle Leclerc), with approval from the local ethical committee (Animal Welfare and Ethics Review Board, AWERB), at the Central Biological Services (CBS) unit at the Hammersmith Campus of Imperial College London. Animals were euthanized at the end of the study (i.e. immediately after the completion of the indicated protocols) by cervical dislocation followed by termination of the circulation by decapitation. The study used a total of 42 experimental animals.

#### Oral (OGTT) and intraperitoneal (IPGTT) glucose tolerance tests

Mice were fasted overnight (15 h) and given free access to water. Glucose (1 g/kg body weight) was administered via either oral gavage or intraperitoneal injection. Blood was sampled from the tail vein at 0, 15, 30, 45, 60, 90 and 120 min. after glucose administration. Blood glucose was measured with an automatic glucometer (Accuchek; Roche, Burgess Hill, UK).

#### Plasma glucagon and insulin measurement

For measurement of glucagon and insulin levels *in* vivo, 100μl of blood was collected from the tail vein into heparin-coated tubes (Sarstedt, Beaumont Leys, UK). Plasma was separated by sedimentation at 10,000 ***g*** for 20 min (4°C). Plasma glucagon or insulin levels were measured in 50 μL aliquots by radioimmunoassay with competitive ^125^I-labelled glucagon or insulin (Millipore, Watford, UK).

#### Measurement of plasma GLP-1

Mice were fasted overnight (15 h), or fed *ab libitum*, and 50μl blood collected from the tail vein into EDTA-coated tubes. Plasma was separated by centrifugation at 4°C, 13,200 r.p.m. for 20 min. and total GLP-1 levels assayed using a two-site microtitre plate-based immunoassay with electrochemical luminescence detection (Meso Scale Discovery Kit, Gaithersburg, MD).

#### Insulin tolerance tests

Mice were fasted for 5 h with free access to water before intraperitoneal injection with 0.75 U/kg bovine insulin (Sigma, Dorset,UK). Blood glucose levels were measured at 0, 15, 30, 45 and 60 min.

#### Immunohistochemistry of pancreatic and intestinal sections

Isolated pancreata and sections of the ileum were fixed in 10% (vol/vol) buffered formalin and embedded in paraffin wax within 24 h of removal. Details of the subsequent immunohistochemical analysis are provided under S2 File (Supplementary Methods).

#### Pancreatic islet isolation

Islets were isolated as described previously [36]. Further details are provided under S2 File (Supplementary Methods).

#### Glucagon secretion

Glucagon secretion was measured from groups of 12 size-matched islets per well essentially as described [30]. Further details are provided under *Supplementary Methods*.

#### Statistics

Data were analysed using GraphPad PRISM 6.0 software. Significance was tested using unpaired Student’s two-tailed t-tests with Bonferroni post-tests for multiple comparisons, or two-way ANOVA as indicated. P<0.05 was considered significant and errors signify ± SEM.

## Results

### Deletion of the AMPK α-subunits improves oral but not intraperitoneal glucose tolerance

**Fig 1:**
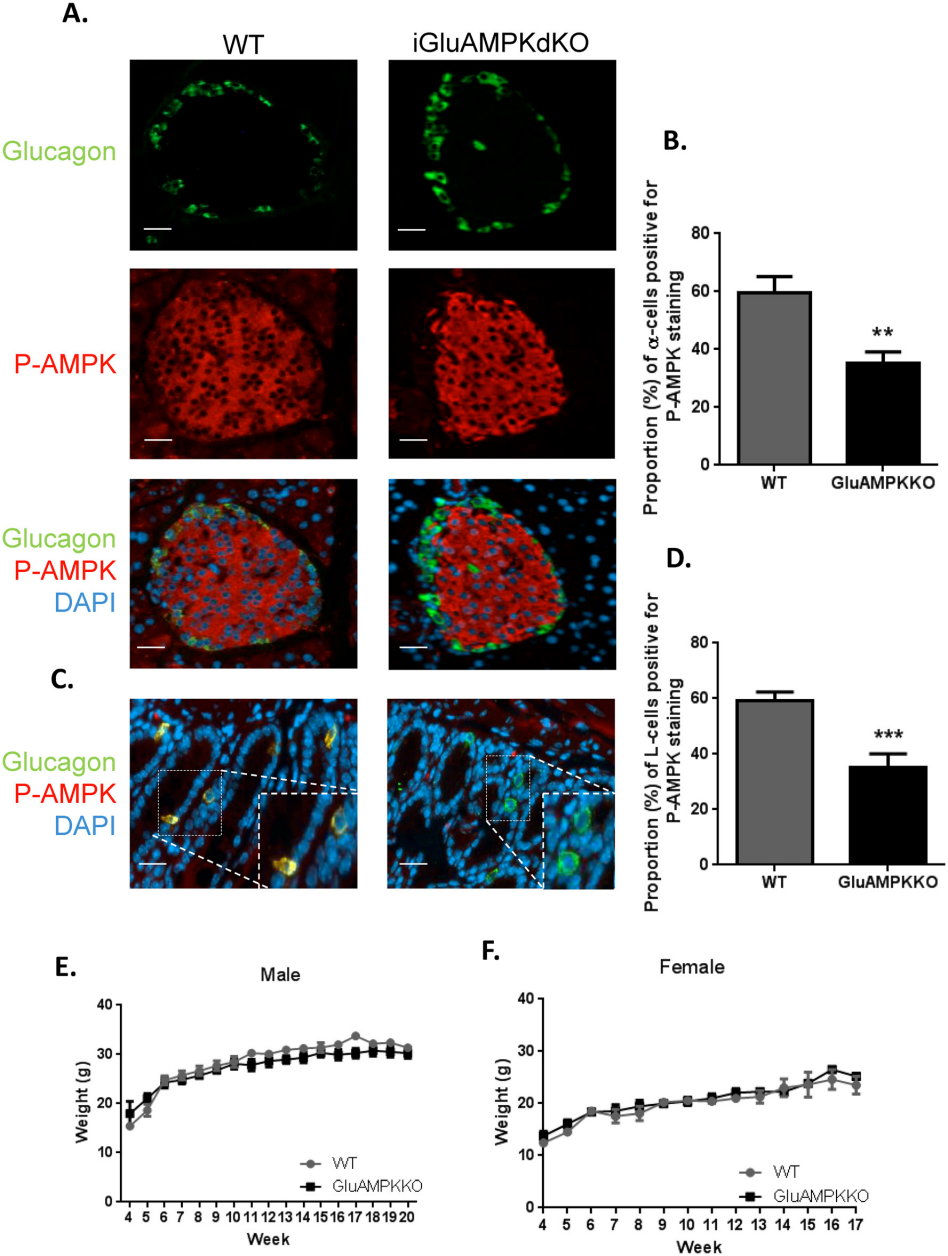
AMPK is deleted selectively in pancreatic α-cells and enteroendocrine L-cells in iGlu AMPKdKO mice. (A) Immunofluorescent staining of pancreatic islets using P-AMPKα1/α2 (red) and proglucagon (green) antibody in iGluAMPKdKO mice *versus* WT littermates, (B) percentage of α-cells co-staining for AMPK and glucagon. (C) Immunofluorescent staining of ileum slices staining with the above antibodies to quantify AMPK knock-down in enteroendocrine cells (D), *n =* 3 mice/genotype (all male). Body weight was measured weekly in (E) male and (F) female iGluAMPKdKO and WT mice, weeks 4–20. *N =* 9–11 mice/genotype, **P<0.01, ***P<0.001, by unpaired Student’s t-test. Data are expressed as means ± SEM.

Fig 1A demonstrates the deletion of AMPK from pancreatic alpha-cells in iGluCre:AMPKα1:α2KO (subsequently referred to as iGluAMPKdKO) mice by immunocytochemical analysis. The percentage of alpha-cells co-stained with anti-P-AMPK α1/α2 and anti-glucagon antibodies was calculated (Fig 1B) and revealed a ~2-fold decrease in P-AMPK levels in the null mice as assessed in terms of the proportion of pAMPK immuno-positive cells (WT: 59.6% ± 5.57, KO 35.1% ± 4.04, P<0.01). Pancreatic slices were also co-stained for P-AMPK and insulin demonstrating that AMPK was not deleted in beta-cells (S1 Fig, S1 File, WT; 83.5% ± 5.19, KO; 76.8 ± 2.07, P>0.05).

Both AMPKα isoforms were detected at the mRNA level in purified wild-type L-cells across the intestine, with α1 predominating (S2 Fig). By immunocytochemical analysis of sections of the ileum, P-AMPK was detected in a smaller proportion of L-cells from iGluAMPKdKO mice (P<0.001) *versus* WT littermates (35.3% ± 4.75 and 59.3% ± 2.95, respectively, Fig 1C and 1D), consistent with recombination in the majority of L-cells.

Body weight was measured weekly in WT and KO mice from weaning (4 weeks) until 20 weeks of age in males, and 17 weeks of age in females (Fig 1E and 1F) and revealed no differences between genotypes. iGluAMPKdKO mice appeared healthy and were not bloated in appearance, in contrast to LKB1 KO mice produced using the same proglucagon promoter-driven *Cre* to achieve recombination of floxed *Lkb1* alleles [33].

**Fig 2:**
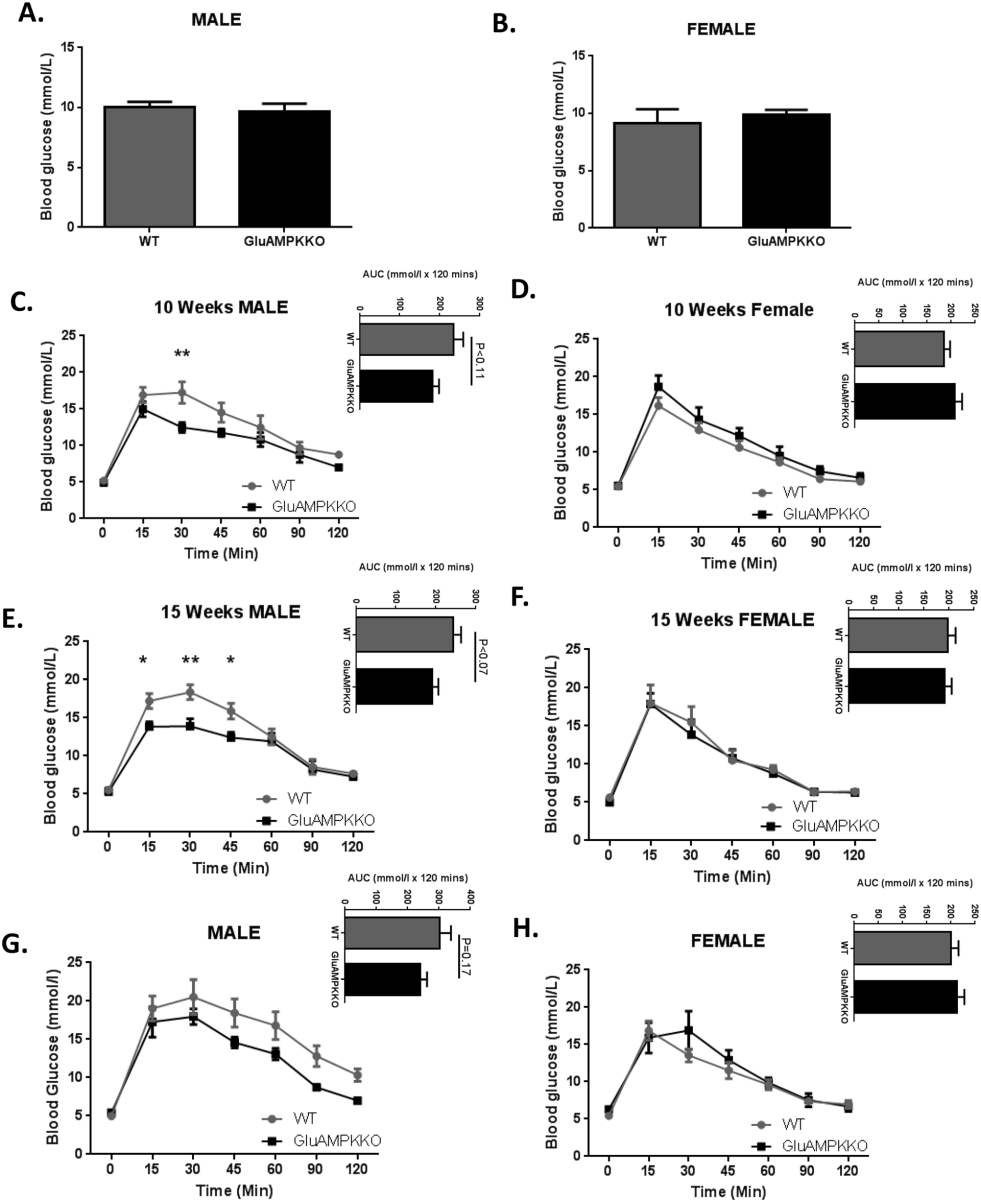
AMPKα1 and -α2 deletion in proglucagon expressing cells improves glucose tolerance after oral administration of glucose in male mice but is unchanged after glucose injection. (A,B) Glycaemia was measured in male and female iGluAMPKdKO and WT mice when fed *ad libitum*. *n =* 6–9 mice/genotype/sex. (C,D) Glucose was administered using oral gavage (1 g/kg) after mice were fasted overnight and blood glucose levels measured at 0, 15, 30, 45, 60, 90 and 120 min. after glucose administration in 10 week old male and female mice. (E, F) Oral glucose tolerance tests repeated in 15 week old male and female iGluAMPKdKO and WT mice. Area under the curve (AUC) is displayed at the top right of panels. (G,H) IPGTTs were performed on 20 week old male and female mice. *n =* 5–9 mice/sex/genotype, *P<0.05, **P<0.01, by 2-way ANOVA. Data are expressed as means ± SEM.

When animals were given free access to food, glycaemia was unchanged in iGluAMPKdKO mice *versus* WT littermates (Fig 2A and 2B). During oral glucose tolerance tests (OGTT), glycaemia was found to be significantly improved in 10 week old male mice at the 30 min. time point (P<0.01) in iGluAMPKdKO mice compared to WT littermates (12.5 ± 0.70 mmol/l and 17.3 ± 1.48 mmol/l, respectively; Fig 2C). Glucose tolerance was enhanced further in 15 week-old male mice 15, 30 and 45 min. after gavage (P<0.05, 0.01, 0.05, respectively) in iGluAMPKdKO mice compared to WT littermates (15 min, 13.8 ± 0.64 *vs* 17.2 ± 0.97 mmol/l; 30 min: 13.9 ± 0.97 *vs* 18.4 ± 0.94 mmol/l and 45 min, 12.4 ± 0.69 *vs* 15.9 ± 1.02 mmol/l, respectively; Fig 2E). Glucose tolerance was unchanged in female KO mice at both 10 and 15 weeks of age during OGTT (Fig 2D and 2F). Intraperitoneal glucose tolerance tests (IPGTTs) on the same cohorts of mice at 20 weeks revealed no significant differences (Fig 2G and 2H).

### AMPKα1 and α2 deletion in proglucagon-expressing cells has no effect on insulin tolerance or circulating insulin levels

**Fig 3:**
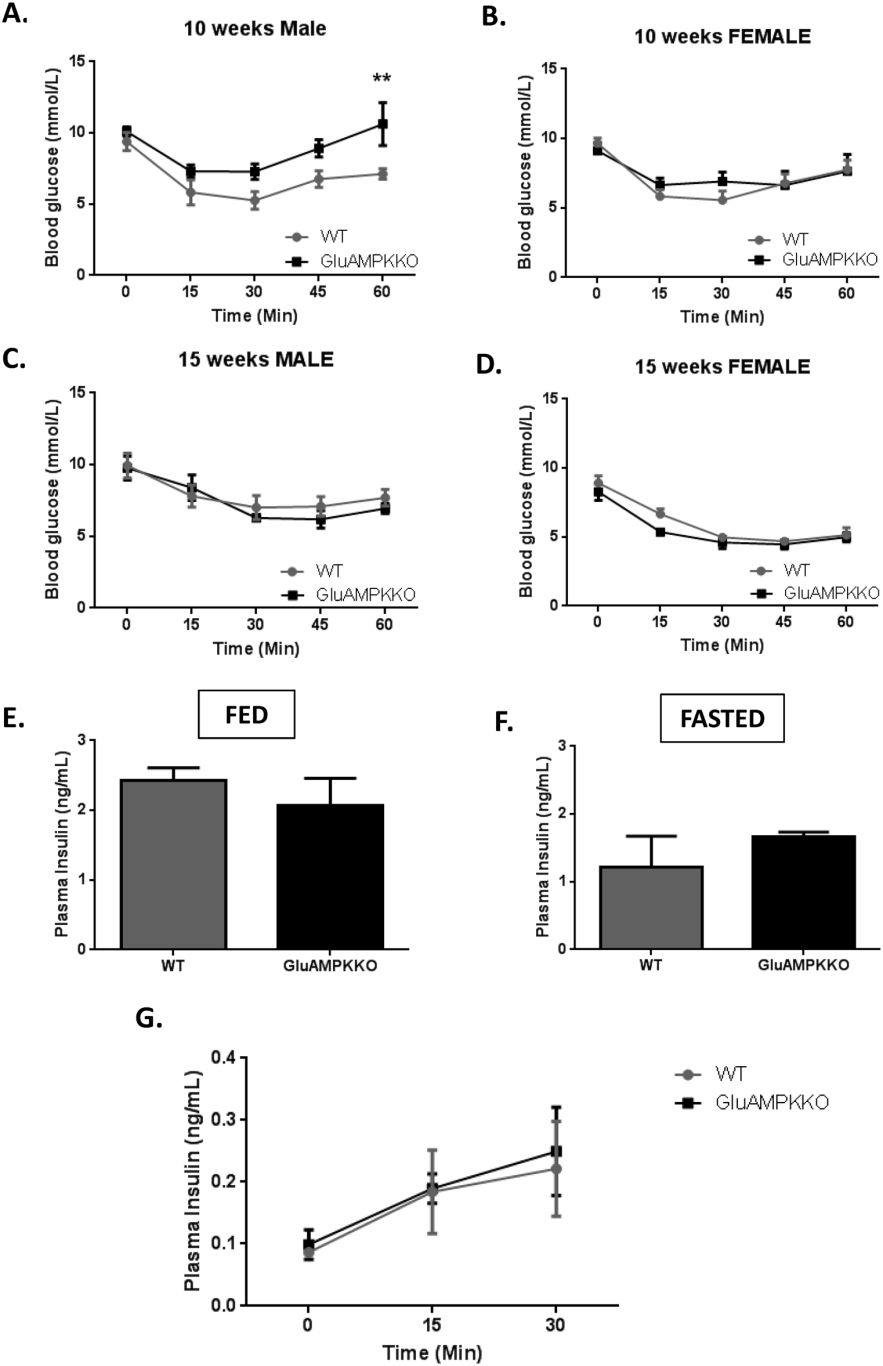
AMPKα1 and -α2 deletion has no or minor effects on glycaemia after insulin injection, or on circulating insulin levels. (A,B) Intraperitoneal insulin tolerance tests (ITTs) were performed on 10 week old male and female mice after mice after fasting for 5 h. Blood glucose levels were measured 0, 15, 30, 45 and 60 min. after insulin injection. (C,D) ITTs were repeated on 15 week old male and female mice, *n =* 5–9 mice/sex/genotype. **P<0.01, by 2-way ANOVA. (E,F) Plasma insulin levels were measured in iGluAMPKdKO and WT mice when fed as normal or after being fasted overnight and (G) at 0, 15 and 30 min. after glucose injection. *n =* 3 mice/genotype, mixed male and female. Data expressed as means ± SEM.

Insulin tolerance tests performed in 10 and 15 week-old male and female mice (Fig 3A–3D) revealed no differences in insulin sensitivity between iGluAMPKdKO mice and their WT littermates except at the 60 min. time point in 10 week-old male mice. Here, blood glucose was significantly higher (P<0.01) in iGluAMPKdKO mice compared to WT animals (10.6 ± 1.52 and 7.12 ± 0.36 mmol/l, respectively), possibly indicating an enhanced counter-regulatory response in the null mice.

Levels of insulin in the plasma of these mice fed *ad libitum* or after overnight fast (Fig 3E and 3F) revealed no changes in insulin levels between iGluAMPKdKO mice and WT littermate controls (Fed: 2.07 ± 0.39 ng/ml, 2.43 ± 0.18 ng/ml, respectively; Fasted: 1.67 ± 0.06 ng/ml, 1.21ng/ml ± 0.46, respectively). Plasma insulin levels were also not different between null and control mice when measured 0, 15 and 30 min. after glucose injection (Fig 3G).

### Deletion of AMPKα1 and α2 in proglucagon-expressing cells results in increased L-cell mass and elevated circulating GLP-1 levels

**Fig 4:**
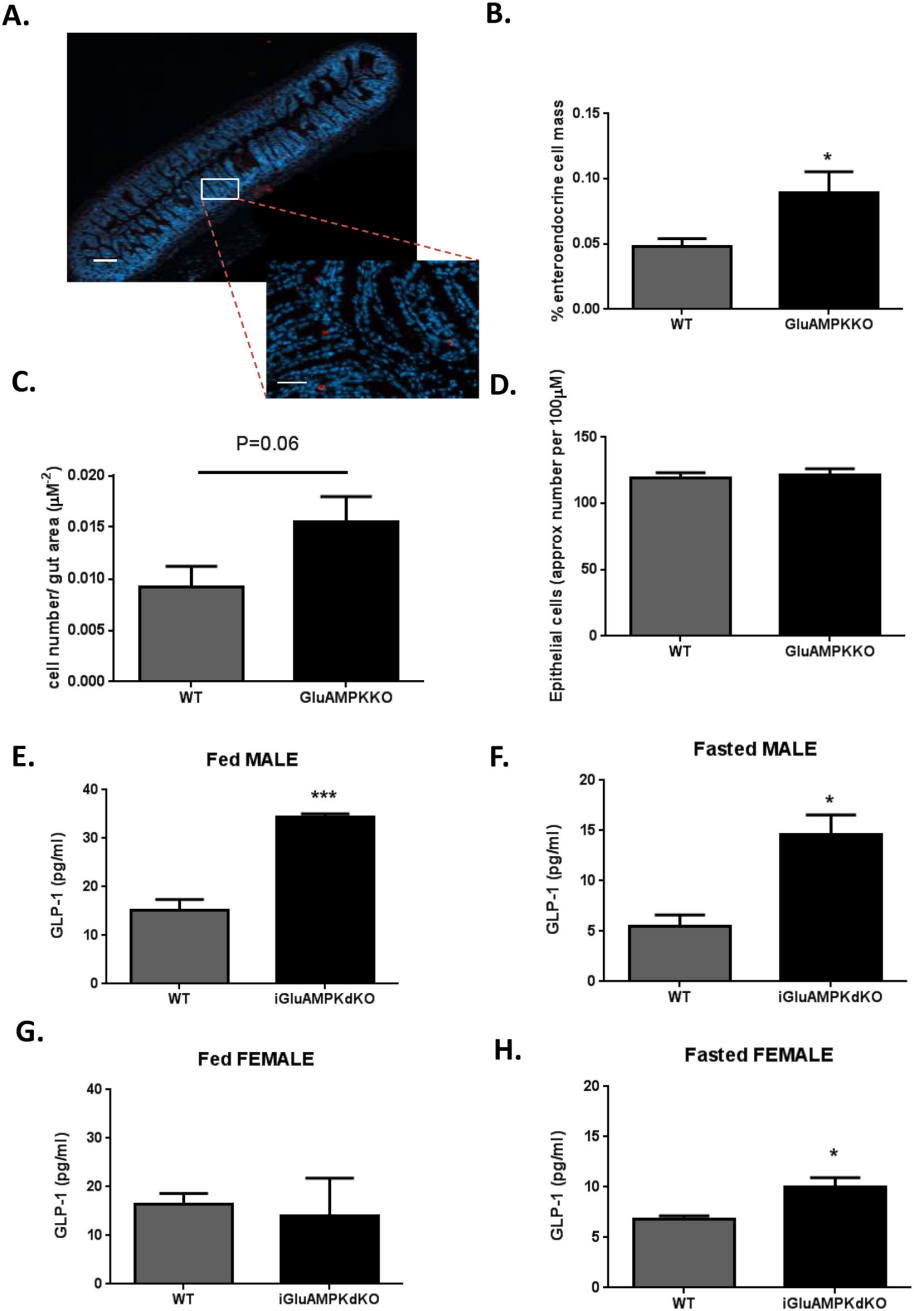
L-cell mass and GLP-1 secretion are enhanced in iGluAMPKdKO mice. (A) Immunohistochemical analysis of an ileum section from an iGluAMPKdKO mouse; red staining represents proglucagon (GLP-1) staining in an enteroendocrine cell, dotted lines represent magnified section. (B) Percentage of proglucagon staining cells in the ileum. (C) L-cell count/area of gut, P = 0.06 by unpaired Student’s t-test. (D) Approximate endothelial cell count for the whole gut section; *n =* 3 mice/genotype (all male). (E,G) GLP-1 levels in the plasma of fed or fasted male and (F,H) female mice, *n =* 3 mice/sex/genotype. *P<0.05, **P<0.01, ***P<0.001 by unpaired Student’s t-test. Data are expressed as means ± SEM.

The improvement in oral (Fig 2B–2E) but not intraperitoneal (Fig 2G and 2H) glucose tolerance is suggestive of an improved incretin response in iGluAMPKdKO mice *versus* controls. To determine whether this may, at least in part, reflect elevated GLP-1 secretion from L-cells in the former, L-cell mass and circulating GLP-1 levels were measured. Sections of the ileum of iGlu AMPKdKO mice and WT littermates were stained with anti-proglucagon antibody, allowing the proportion of proglucagon-expressing cells to be determined (Fig 4A and 4B). This approach revealed an 80% increase in overall L-cell mass in male iGluAMPKdKO (0.09% ± 0.02) *versus* WT littermates (0.05% ± 0.01, *n =* 3 mice/genotype, 5 slides/mouse and 15–30 islets counted/slide; P<0.05). A tendency towards an increase in individual cell number per unit area (0.015 ± 0.002 for KO and 0.009 ± 0.002 μm^-2^ for WT, p = 0.06, *n* = 3 animals/genotype) was also apparent (Fig 4C). No differences in overall epithelial cell number/unit area, as estimated through nuclear staining, were detected (Fig 4D).

Plasma GLP-1 levels were also measured in iGluAMPKdKO mice and littermate controls and were significantly elevated in male KO mice versus controls both after *ad libitum* feeding (KO, 34.33 ± 0.72 pg/ml *versus* WT, 15.1 ± 2.25 pg/ml, *n =* 3 mice/ genotype, p<0.001, Fig 4E) or after overnight fast (KO 14.6 ± 1.95 pg/ml *vs* WT 5.47 ± 1.17 pg/ml, Fig 4F). By contrast, for female mice, no differences between iGluAMPKdKO and control animals were observed in the fed state (KO, 13.9 ± 7.83 pg/ml *versus* WT, 16.4 ± 2.19 pg/ml, *n =* 3 mice/genotype, p>0.05, Fig 4G) whereas a modest elevation in plasma GLP-1 was measured in the fasted state (KO, 10.0 ± 0.88 pg/ml *vs* WT, 6.77 ± 0.37 pg/ml, respectively, p<0.05, Fig 4H).

### AMPKα1, -α2 deletion in pancreatic alpha-cells has no effect on alpha-cell mass or glucagon secretion

**Fig 5:**
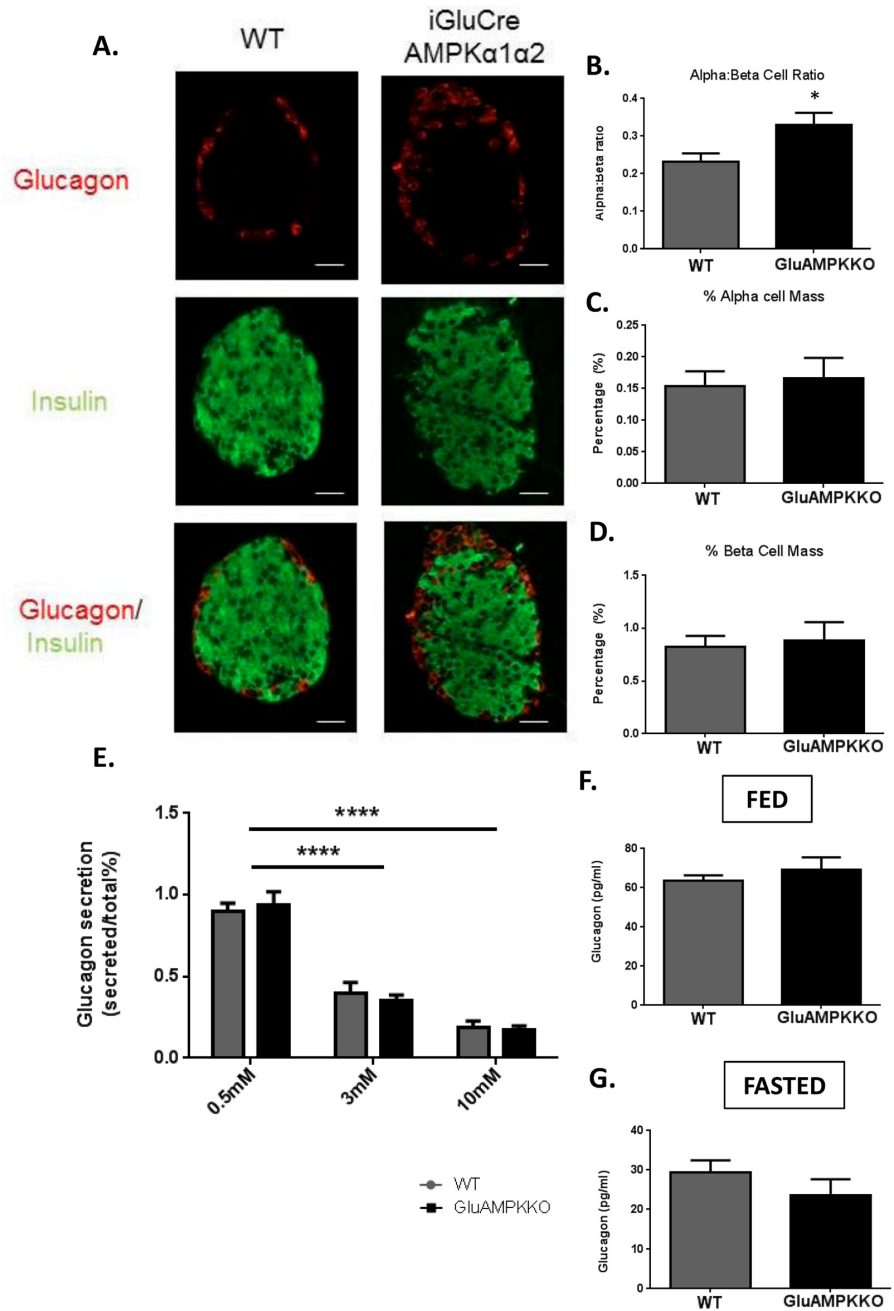
AMPKα1,α2KO has no effect on α-cell mass or glucagon secretion in male mice. (A) Immunofluorescent staining of pancreatic sections using guinea pig anti-insulin (1:200; green) or rabbit anti-glucagon (1:100; red) antibodies from iGluAMPKdKO or WT mice. (B) Alpha:beta cell ratio, (C) % alpha-cell mass, (D) % beta-cell mass; *n =* 3 mice/genotype (all male), (E) glucagon secretion, measured from 12 size-matched islets incubated in 0.5, 3 or 10 mmol/l glucose and measured using radioimmunoassay, (F,G) levels of glucagon in the plasma of iGluAMPKdKO and WT mice when fed or fasted overnight. *n =* 6 mice/genotype, *P<0.05, by Student’s t-test; ****P<0.0001 by 2-way ANOVA. Data are expressed as means ± SEM.

To investigate whether AMPKα1 and α2 play a role in pancreatic islet cell growth or survival, pancreatic sections from male mice were co-stained with insulin and glucagon in order to measure pancreatic beta- and alpha-cell mass, respectively, as well as the alpha:beta cell ratio (Fig 5A–5D). Though the alpha:beta cell ratio was increased in AMPKα1α2KO mice *versus* WT littermates (P<0.05), no changes were found in overall alpha- or beta-cell mass.

We next determined whether deleting the AMPKα subunits in pancreatic alpha-cells might affect glucagon secretion *in* vitro. Here, isolated islets were incubated in 0.5, 3 or 10 mmol/l glucose (Fig 5E). Similar to alpha-cell mass, AMPKα1/α2 deletion exerted no effect on glucagon secretion. Plasma glucagon levels were measured in fed and fasted mice (Fig 5F and 5G) and, consistent with earlier results [30] using an alternative proglucagon promoter, were unaffected in iGluAMPKdKO mice in comparison to WT littermates.

## Discussion

The first aim of this study was to determine whether deleting AMPKα1 and α2 in proglucagon-expressing cells would phenocopy the effect of deleting LKB1 in these cells using the same iGluCre transgene [33] and produce large gastro-duodenal PJS like-polyps. In marked contrast to our earlier findings [33], iGluAMPKdKO mice showed no signs of tumour development or premature death, suggesting that members of the AMPK-related kinase (AMPK-RK) family [32], rather than canonical AMPK α1 or α2-containing complexes, are responsible for the suppression of polyposis from proglucagon-expressing cells [37]. Possible candidates include Par1b/MARK2 [38], which controls cell polarity and is implicated in beta cell hyperplasia after LKB1 deletion [39,40], as well as NUAK1/SNARK [41] and NUAK2 [42].

### Deletion of AMPKα1 and α2 in L-cells improves GLP-1 secretion and glucose tolerance

In further contrast to the effects of deleting LKB1 in proglucagon-expressing cells using the iGluCre deleter strain, male mice lacking both AMPK catalytic subunits in these cells, including enteroendocrine L-cells, displayed improved oral glucose tolerance (Fig 2C and 2E). Interestingly, glucose tolerance during IPGTT was not significantly affected in iGluAMKdKO mice (Fig 2G and 2H), although it should be noted that the latter tests were performed in the same animals at a slightly later stage (at 20 *vs* 15 weeks of age). These findings suggest that the incretin response is enhanced in the iGluAMPKdKO model. Providing direct evidence for this view, GLP-1 release into the plasma was significantly increased in iGluAMPKdKO mice, as assessed in fed and fasted mice (Fig 4E–4H). Although we were not able to detect differences in fasting or fed plasma insulin levels between WT and KO mice (Fig 3E and 3F), nor altered plasma insulin during IPGTTs (Fig 3G), lower glucose levels were observed in male mice during OGTTs (Fig 2C and 2E) and are indicative of enhanced beta cell function, as expected given elevated circulating GLP-1 levels.

We have also considered the possibility that the above observations might suggest a local action of GLP-1 on enteric neurons close to the site of release which then initiates an paracrine loop to regulate hepatic glucose production [43]. However, in the latter study the acute action of metformin, an AMPK activator, in the duodenum was to *reduce* hepatic glucose production via an AMPK-dependent mechanism. Metformin has also previously been found to increase plasma active GLP-1 levels in wild-type rats [44], as well as in rats lacking dipeptidyl peptidase IV (DPPIV) [45], and to enhance GLP-1 secretion from a human-derived intestinal L-cell line (NCI-H716) [46]. These observations argue for an acute stimulation of GLP-1 release in response to the biguanide. The apparent contrast between the present study and earlier findings [43] [45] may thus suggest that metformin acts (1) on GLP-1 secretion independently of AMPK, as recently reported in studies of hepatic gluconeogenesis [47], or (2) that there may be significant differences between the impact of acute and chronic activation of L-cell AMPK on a “gut-brain-liver axis”, with the latter acting chiefly to alter cell mass.

Interestingly, the above changes in oral glucose tolerance were apparent in male but not female mice and reflected more minor changes in circulating GLP-1 levels. Whilst the reasons for these differences must remain speculative, they may include greater variance in the measurements for females (e.g. due to reproductive cycles), differences in feeding patterns or sexual dimorphism in the expression of GLP-1, insulin or other receptors.

### AMPKα1 and α2 deletion from pancreatic alpha-cells does not alter alpha-cell mass or glucagon secretion but increases L-cell mass

In the present study no changes in alpha- or beta-cell mass were observed in iGluAMPKdKO mice consistent with previous findings using the shorter glucagon promoter-*Cre* to drive deletion in these cells [30]. Nonetheless, and reflecting the greater statistical power to detect small differences, we did observe a small increase in alpha to beta cell ratio in the null mice, suggesting some degree of hyperplasia (or hypertrophy) of alpha cells (or a decrease in in beta cell number/mass). Future studies, on larger cohorts, will be needed to explore these findings in more detail and might be supported by measurements of hepatic glucose output or pyruvate tolerance as further estimates of glucagon action.

AMPK is a regulator of the mTOR complex, that goes on to phosphorylate the mTORC1 components Raptor and TSC2 [48] which are involved in the regulation of cell size [49] and proliferation. Interestingly, a clear increase in total L-cell mass, expressed as a fraction of intestinal surface, was apparent (Fig 4A and 4B) alongside a strong tendency towards an increase, of similar magnitude, in the number of L-cells per unit area. Future investigations will be required to explore the mechanisms involved, which might conceivably include the activation of mTORC1 as a result of AMPK deletion. Additionally, Carbohydrate Response Element Binding Protein (ChREBP), a transcriptional regulator of proglucagon gene expression and carbohydrate metabolism in L-cells [50], is also inhibited by AMPK [51], and may further increase GLP-1 synthesis and secretion.

Consistent with previous findings [30] in which both AMPK α1 and α2 were deleted in the alpha-cell using a shorter (1.6 kB) preproglucagon promoter [52] to drive *Cre* expression, we obtained no evidence for changes in circulating glucagon levels after ablation of AMPK activity. Of note, the longer proglucagon promoter used in the present study deletes additionally in enteroendocrine L- and probably other proglucagon-expressing cells throughout the body (e.g. in the brain stem and olfactory bulb) [35]. Thus, we show that deleting both AMPK α-subunits in pancreatic alpha-cells has little effect on glucagon release in response to low glucose levels *in vitro* or *in vivo*. Likewise, in the iGluAMPKdKO mice, glucagon secretion was not enhanced in iGluAMPKdKO mice *versus* controls during hypoglycaemia induced by insulin injection (results not shown). It should be noted, however, that when AMPK α1, but not α2, was deleted selectively in the alpha-cell with the shorter proglucagon promoter-driven Cre, impaired glucagon secretion was observed both *in vivo* during hypoglycemic clamp and *in vitro* [30]. Furthermore, AMPK activation was previously found to regulate glucagon secretion *in vitro* in a study using clonal αTC1-9 cells [29]. In the latter study, AMPK expression was stimulated using pharmacological agents such as metformin, and resulted in enhanced glucagon secretion at both high and low glucose concentrations. One possible explanation for the apparent discrepancy between the present and earlier studies [30] is that complete inactivation of AMPK in the α-cell *in vivo* leads to more robust compensatory changes than seen after AMPKα1 deletion alone.

### Conclusions

Whilst not required prevent polyposis [33], AMPK limits expansion of the L-cell population and GLP-1 release, processes which may provide a new therapeutic avenue for the treatment of T2D.

## Supporting Information

S1 Fig AMPK expression is unaffected in β-cells from iGluAMPKdKO mice. (TIFF)

S2 Fig Ampkα1 and -α2 gene expression in purified L-cells and control non-fluorescent cells from the upper small intestine, lower small intestine and colon. (TIFF)

S1 File Supplementary Figure Legends. (DOCX)

S2 File Supplementary Methods. (DOCX)

## Abbreviations

AMPK: AMP-activated protein kinase
EMT: epithelial-to-mesenchymal transition
GLP-1: glucagon-like peptide-1
GLP1R: GLP-1 receptor
iGluCre: preproglucagon promoter-driven *Cre* recombinase
ITT: insulin tolerance test
IPGTT, OGTT: intraperitoneal or oral glucose tolerance test
LKB1: liver kinase B1
