# Supplementary figures and images for: Proglucagon Promoter Cre-Mediated AMPK Deletion in Mice Increases Circulating GLP-1 Levels and Oral Glucose Tolerance
Source: PLoS One. 2016 Mar 24;11(3):e0149549. doi: 10.1371/journal.pone.0149549 (PMC4806996; doi:10.1371/journal.pone.0149549)

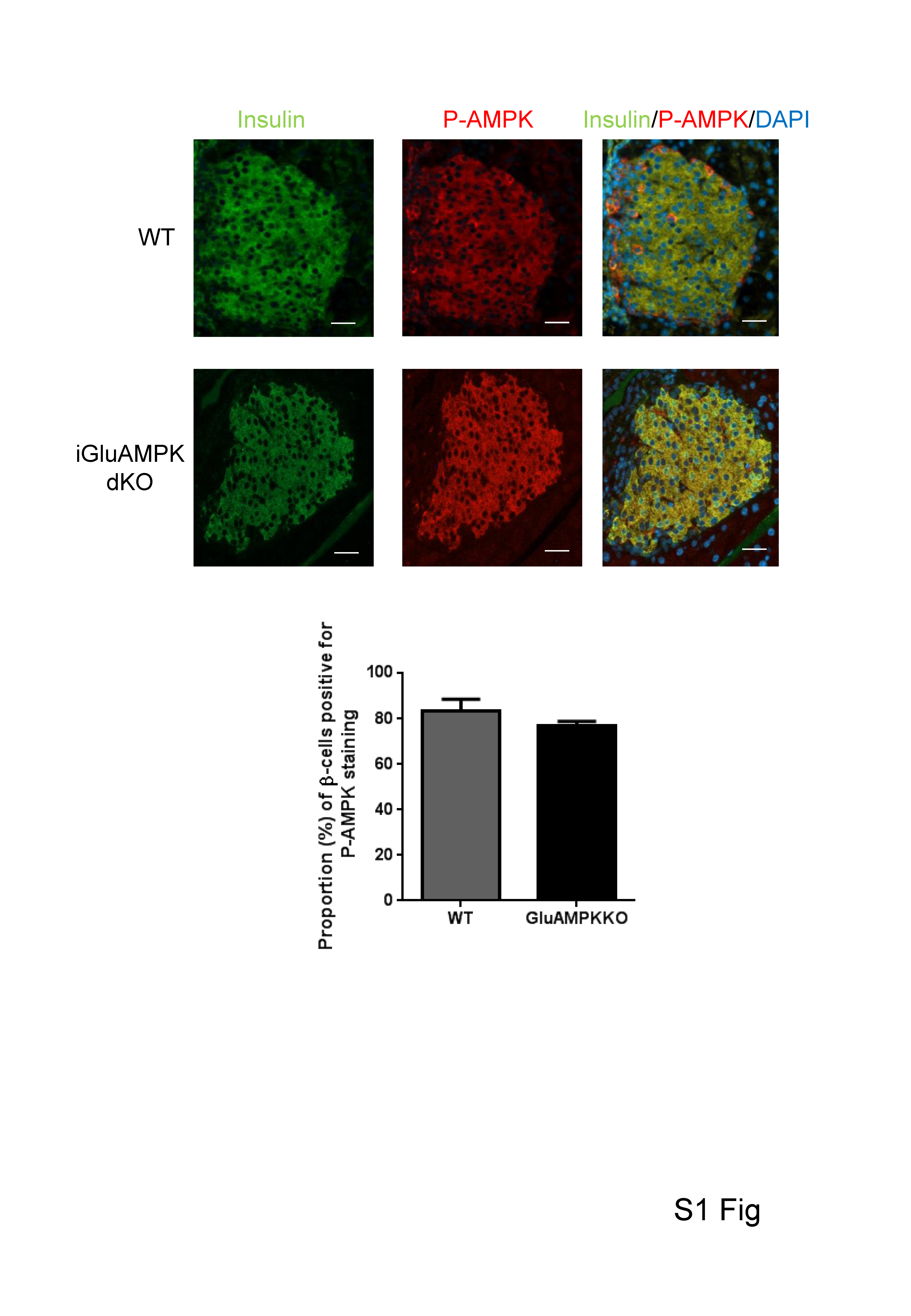

Supplement: S1 Fig — (TIFF) [file pone.0149549.s001.tiff]

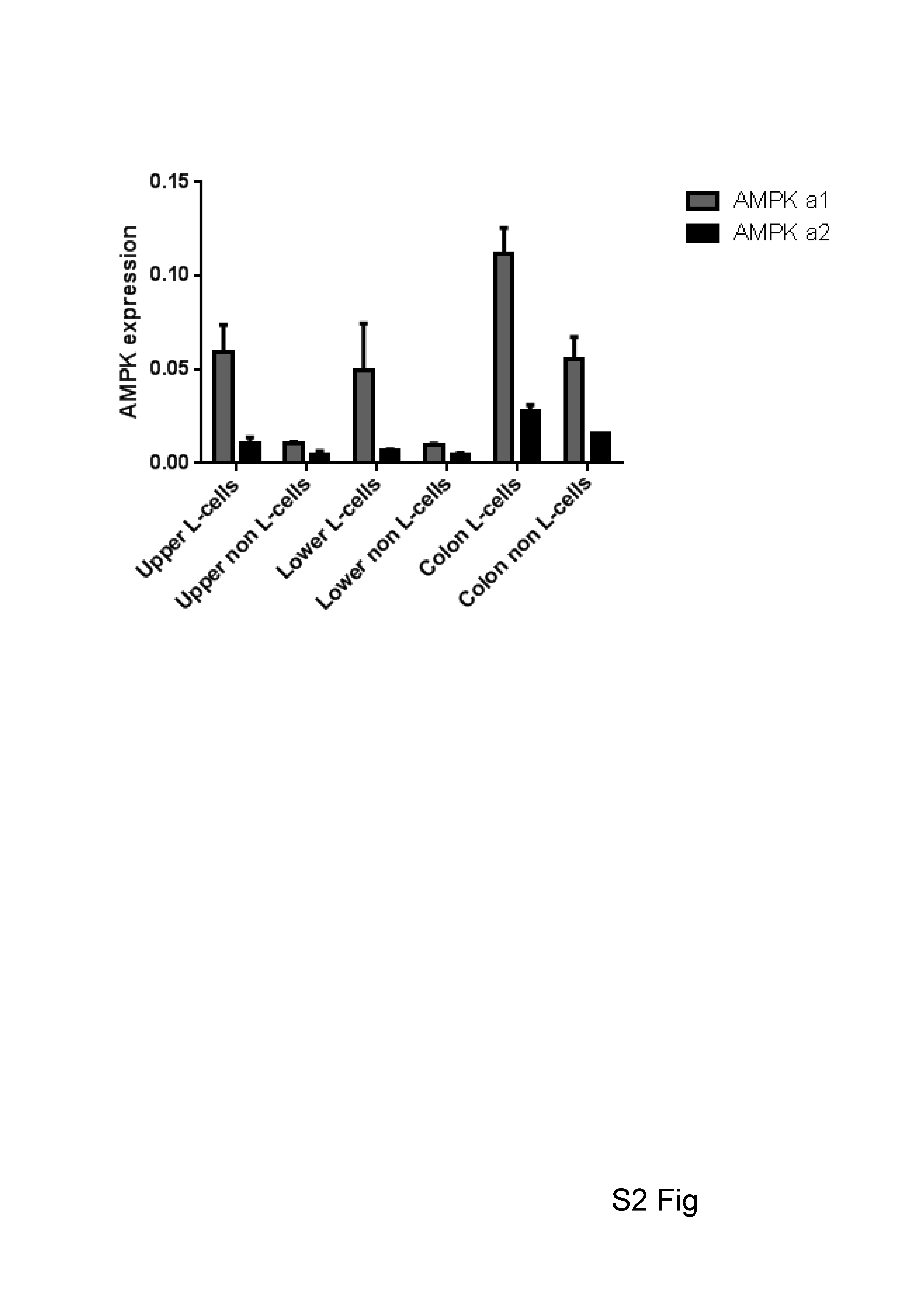

Supplement: S2 Fig — (TIFF) [file pone.0149549.s002.tiff]
