# Supplementary material for: Proglucagon Promoter Cre-Mediated AMPK Deletion in Mice Increases Circulating GLP-1 Levels and Oral Glucose Tolerance
Source: PLoS One. 2016 Mar 24;11(3):e0149549. doi: 10.1371/journal.pone.0149549 (PMC4806996; doi:10.1371/journal.pone.0149549)
Supplement: S1 File — (DOCX) [file pone.0149549.s003.docx]

S1 File. Supplementary Figure Legends

S1 Fig. **AMPK expression is unaffected in β-cells from iGluAMPKdKO mice**

(A) Staining of pancreatic sections using the anti-rabbit P-AMPKα1α2 antibody (red) and anti-guinea-pig insulin antibody (1:200; green) antibodies to quantitate the % of β-cells co-stained for insulin and AMPKα1 (B). *N*= 3 mice/genotype, data are expressed as means ± SEM.

S2 Fig. **Ampkα1 and -α2 gene expression in purified L-cells and control non-fluorescent cells from the upper small intestine, lower small intestine and colon.**

Expression was determined after purification of the indicated cell types from mice over-expressing yellow fluorescent protein selectively in L-cells under the preproglucagon promoter. Purification was by fluorescence-activated cell sorting [[1](#_ENREF_1)]. Quantification was by quantitative RT-PCR. The sequences used to amplify Ampkα1 (*Prkaa1*; grey bars) and Ampkα2 (*Prkaa2*, black bars) were as follows: -α1 forward: TGATGCCGAAGCTCAAGGA; -α1 reverse: GGGAGGTGACAGAGGTAAGG; -α2 forward: AACATGGGCGGGTTGAAGA; -α2 reverse: ATCCACGGCAGACAGGATCT. Cyclophilin was used as a control for normalisation. Data are expressed as means ± SEM; n = 2-3 mice per genotype.

[1] Reimann F H.A., Tolhurst G, Parker HE, Rogers GJ, Gribble FM, 2008. Glucose sensing in L cells: a primary cell study. Cell Metab 8(6): 532-539
