# Supplementary material for: Proglucagon Promoter Cre-Mediated AMPK Deletion in Mice Increases Circulating GLP-1 Levels and Oral Glucose Tolerance
Source: PLoS One. 2016 Mar 24;11(3):e0149549. doi: 10.1371/journal.pone.0149549 (PMC4806996; doi:10.1371/journal.pone.0149549)
Supplement: S2 File — (DOCX) [file pone.0149549.s004.docx]

S2 File. Supplementary Methods**.**

## Pancreatic islet isolation.

Pancreata were inflated with 1mg/ml collagenase solution (Serva) and incubated after removal in a water bath for 10 min at 37°C. Ice-cold additive-free RPMI (Sigma) was added and centrifuged and supernatant removed before repeating this step 3X. Islets were re-suspended in 3ml Histopaque-119 (Sigma) and a sucrose gradient created using Histopaque-1083 (Sigma) and RPMI. The gradient was centrifuged at 2500rpm for 20 min., revealing an islet layer just below the RPMI layer. Islets were collected and resuspended in RPMI which was once again centrifuged and the supernatant removed. Islets were re-suspended in RPMI media containing additives and, after recovery at 37°C, were handpicked and placed in fresh medium.

## Glucagon secretion.

Secretion was measured from 12 size-matched islets per condition. Islets were initially pre-incubated in 1.0 ml modified Krebs-HEPES bicarbonate buffer (in mmol/l: 130 NaCl, 3.6 KCl, 1.5 CaCl_2_, 0.5 MgSO_4_, 0.5 KH_2_PO_4_, 2 NaHCO_3_, 10 HEPES, and 0.1% (w/v) BSA, pH 7.4, equilibrated for 20 min with O_2_/CO_2_ (95:5 (v/v)) at 37 °C with 10 mmol/l glucose at 37°C with constant agitation for 1 h. This was followed by supplementation of the medium with either 0.5, 3 or 10 mmol/l glucose (final volume 500 µl) and incubation for a further 30 min. Total islet glucagon was extracted from islets in acidified ethanol (75 % (v/v) ethanol, 1.5 mmol/l HCl, 0.1% (w/v) Triton) by sonication with an ultrasonic converter for 60 s. Secreted and total glucagon were measured using ^125^I labelled glucagon by radio-immunoassay (Millipore).

## Immunohistochemistry of pancreatic and intestinal sections

Isolated pancreata and sections of the ileum were fixed in 10 % (vol/vol) buffered formalin and embedded in paraffin wax within 24 h of removal. Sections were cut at 5 μm (pancreas) and 7 μm (ileum) and incubated overnight at 37°C on superfrost slides. Slides were submerged sequentially in Histoclear (Sigma) followed by washing in decreasing concentrations of ethanol to remove paraffin wax. Permeabilised pancreatic slices were blotted with anti-rabbit P-AMPKα1/α2 (Cell Signalling tech), anti-guinea pig insulin (Dako, Ely, UK) or rabbit anti- human glucagon/GLP-1 (Santa Cruz rabbit polyclonal FL-180/sc-13091; recognises amino acids 1-180 of full length proglucagon, including the GLP-1 sequence, 72-107/8) as primary antibodies, and were visualised by subsequent incubation with Alexa Fluor 488-labelled donkey anti-rabbit, Alexa Fluor 488-labelled goat anti-guinea pig or Alexa Fluor 568-labelled donkey anti-rabbit antibodies. Samples were mounted on glass slides using Vectashield^TM^ (Vector Laboratories) and hard set with DAPI. Images were captured on a Zeiss Acio Observer.Z1 Motorised inverted widefield microscope fitted with a Hamamatsu Flash 4.0 Camera using a Plan-Apochromat 206/0.8 M27 air objective with Colibri.2 LED illumination. Data acquisition was controlled by Zeiss Zen Blue 2012 Software. Fluorescent quantification was achieved using Image J. Ratios were calculated using an in-house macro for comparative analysis between sample groups. Whole pancreas or ileum area was used to quantitate cell mass.
